# Predictors of *Toxoplasma gondii* IgG Seropositivity and Cranial Ultrasound Patterns among Children with Hydrocephalus

**DOI:** 10.1155/2020/8326348

**Published:** 2020-09-04

**Authors:** Sofia Ottaru, Mariam M. Mirambo, Rogatus Kabyemera, Benson R. Kidenya, Mwanaisha Seugendo, Delfina R. Msanga, Patrick Ngoya, Domenica Morona, Stephen E. Mshana

**Affiliations:** ^1^Department of Paediatrics & Child Health, Bugando Medical Centre, P.O. Box 370, Mwanza, Tanzania; ^2^Department of Microbiology and Immunology, Weill Bugando School of Medicine, Catholic University of Health and Allied Sciences, P.O. Box 1464, Mwanza, Tanzania; ^3^Department of Biochemistry and Molecular Biology, Weill Bugando School of Medicine, Catholic University of Health and Allied Sciences, P.O. Box 1464, Mwanza, Tanzania; ^4^Department of Radiology, Bugando Medical Centre, P.O. Box 370, Mwanza, Tanzania; ^5^Department of Medical Parasitology and Entomology, Weill Bugando School of Medicine, Catholic University of Health and Allied Sciences, P.O. Box 1464, Mwanza, Tanzania

## Abstract

**Background:**

*Toxoplasma gondii* infection during pregnancy is associated with serious neonatal complications, including hydrocephalus. In many high-income countries, *T. gondii* screening and treatment during the antenatal period are routinely carried out to prevent associated complications, whereas in most low-income countries, there is no routine screening of *T. gondii* during pregnancy. Despite the parasite being common in Tanzania, there is a paucity of information on the prevalence of *T. gondii* and cranial ultrasound patterns among children with hydrocephalus.

**Methods:**

An analytical cross-sectional hospital-based study involving 125 infants with hydrocephalus attending the Bugando Medical Centre (BMC) was conducted between May 2017 and February 2018. Sociodemographic and other relevant information was collected using a pretested data collection tool. Venous blood samples were collected, and sera were used for the detection of specific *T. gondii* antibodies by indirect enzyme-linked immunosorbent assay (ELISA) as per manufacturer's instructions. Data were analysed using STATA version 13 software.

**Results:**

The mean age of enrolled children was 4.8 ± 3.5 months. Out of 125 infants with hydrocephalus, 29 (23.2%, 95% CI: 21-36) were seropositive for *T. gondii*-specific IgG antibodies. By multiple generalized linear model analysis, being male (aRR = 1.1, 95% CI: 0.9–1.5, *p* = 0.049), higher birth order (aRR = 1.2, 95% CI: 1.0–1.5, *p* = 0.023), consumption of fish meat (aRR = 1.6, 95% CI: 1.2–2.3, *p* = 0.003), and using other methods of cooking meat than boiling (aRR = 1.7, 95% CI: 1.1–2.5, *p* = 0.015) were independent risk factors for *T. gondii* IgG seropositivity. Obstructive hydrocephalus was significantly more common among *T. gondii-*seronegative infants compared to IgG-seropositive infants (31.3% [30/96] vs. 13.8% [4/29]; *p* = 0.049).

**Conclusions:**

A significant proportion of infants with nonobstructive hydrocephalus are *T. gondii* IgG seropositive, and this is predicted by male gender, increase of birth order, consuming fish, and using other methods of cooking meat than boiling. These facts highlight the importance of continuing health education for pregnant women regarding *T. gondii* transmission and the need to follow-up their infants so that appropriate counselling and management can be provided.

## 1. Background

*Toxoplasma gondii* infection is a public health problem with detrimental effect to the developing fetus. The mother-to-child transmission risk of *T. gondii* infection is estimated to be 29%, and seropositivity has been found to increase with an increase in age and gestation age [1–4]. Acquisition of this infection during pregnancy, especially in the first trimester, can lead to serious neonatal complications which include retinochoroiditis, hydrocephalus, intracranial calcifications, and convulsions [5].

Hydrocephalus (HDC) is a disturbance of the cerebrospinal fluid (CSF) formation, flow, or absorption, leading to a high volume of CSF in the central nervous system (CNS) [6]. About 750,000 children and adults are living with hydrocephalus, and one in every 500 live births is affected worldwide [7]. In high-income countries (HIC), the incidence of congenital hydrocephalus has been estimated at 0.5 cases per 1000 live births and the incidence of neonatal hydrocephalus has been estimated at 3-5 cases per 1000 live births [8]. In the East African region, more than 6000 new cases of hydrocephalus occur every year [9].

Congenital toxoplasmosis (CT) has been found to be common particularly when infection occurs in the first trimester; a previous study reported that about 67.7% of children with CT had hydrocephalus, indicating that *T. gondii* is one of the common contributing factors to these cases [10]. The review of the surgical ward admission book at the Bugando Medical Centre (BMC) in 2016 showed that hydrocephalus was the leading cause of admission in children, with at least 15 to 20 new cases admitted every month. Moreover, a previous study by Mashuda et al. reported that 35.9% of the children with congenital malformations had hydrocephalus, making it the second most occurring CNS congenital malformation among infants at BMC [11]. Additionally, previous studies in Mwanza and Dar es Salaam, Tanzania, reported a *T. gondii* IgG seropositivity of about 31% among pregnant women and 55.4% among women with spontaneous abortions [4, 12, 13]; this indicates an increased risk of congenital *T. gondii* infection among unborn children in these settings. Despite a high occurrence of hydrocephalus and a high seroprevalence of *T. gondii* among pregnant women in Mwanza, the association between *T. gondii* seropositivity and hydrocephalus has not been studied in this setting. Likewise, cranial ultrasound patterns among infants with hydrocephalus are not well understood in many centres in developing countries. As a matter of fact, control interventions require the formulation of an evidence-based policy to set guidelines for screening and treating pregnant women in countries where *T. gondii* is endemic. This study is therefore aimed at determining the seropositivity of *T. gondii* antibodies and its predictors and cranial ultrasound patterns among infants with hydrocephalus.

## 2. Methodology

### 2.1. Study Design and Duration

An analytical cross-sectional hospital-based study involving 125 infants aged 0-12 months with hydrocephalus was conducted from September 2017 to April 2018.

### 2.2. Study Site

The study was conducted at the departments of paediatrics and child health and surgery (neurology) of BMC, a referral and teaching hospital for the Lake Victoria and Western Zones of the United Republic of Tanzania. This centre for tertiary specialist care serves eight regions, including Mwanza, Geita, Simiyu, Mara, Kagera, Shinyanga, Tabora, and Kigoma, with a catchment population of approximately 14 million people [14]. The department of paediatrics and child health has a capacity of 121 beds. It is subdivided into general paediatric wards, malnutrition wards, semi-intensive care unit, neonatal unit, neonatal intensive care unit, and outpatient department. Approximately twenty new cases of hydrocephalus are admitted in the surgical departments every month, while in paediatric wards approximately five new cases are admitted every month.

### 2.3. Study Population and Selection Criteria

The study included all infants with hydrocephalus who attended the surgical and paediatric departments of Bugando Medical Centre during the study period. Infants who were critically ill or did not have brain ultrasounds or CT scan results were excluded.

### 2.4. Sample Size and Sampling Procedure

**Figure 1:**
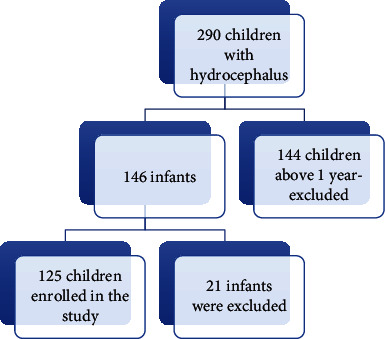
Study enrolment.

For sample size calculation, a formula for comparing two independent sample sizes was used. We assumed the proportion of infants who will be IgG positive to be 8% and those who will be IgM positive to be 0.115%, at 95% confidence interval [15]; the minimum sample size was 125 infants. In a period of 7 months, a total of 290 children with hydrocephalus were attended at BMC, 62 children from the paediatric department and 228 from the surgery department-neurology clinic. A total of 144 children who were above 1 year were excluded from the study. Out of 146 infants who were eligible for the study, 125 (85.6%) infants were conveniently enrolled (Figure 1).

### 2.5. Data Collection Procedure

Detailed history taking and thorough physical examination were performed for all the children enrolled. The information collected included sociodemographic characteristics, maternal details (residency, level of education), and risk factors (like owning a cat, eating undercooked meat, gardening, and drinking unboiled water) associated with *T. gondii* infections. The HIV status of the mother and child's risk factors (like crawling and eating soil) were also noted.

### 2.6. Sample Collection and Laboratory Procedures

Three millilitres (3 ml) of blood was collected from each participant and placed in plain vacutainer tubes (Neomedic Limited, China) for the detection of *T. gondii-*specific IgM and IgG antibodies by using the enzyme-linked immunosorbent assay (ELISA). The blood samples were taken to CUHAS–Bugando multipurpose laboratory where sera were separated from the whole blood by centrifugation at 3000 rpm for 5 minutes. Sera were kept at -40°C until processing. Specific *T. gondii* IgM and IgG antibodies were detected by indirect ELISA (Pishtaz Teb Diagnostics, Tehran, Iran) [16], with sensitivity and specificity for IgM of 100% and 99%, respectively, and sensitivity and specificity for IgG of 100%. All procedures were performed following the manufacturer's instructions and the standard operating procedures of the BMC accredited laboratory.

### 2.7. Cranial Ultrasound Scan

For each study participant, the occipital frontal circumference (OFC) was measured followed by cranial ultrasounds or head CT scan (as an alternative mode of confirmation) to confirm the diagnosis of hydrocephalus. A cranial ultrasound scan was performed, and anatomical patterns of hydrocephalus were denoted as follows: obstruction of the aqueduct of Sylvius, bilateral obstruction of the foramina of Monro, unilateral obstruction of the foramina of Monro, and no obvious intraventricular obstruction causing ventricular dilatation [17]. In addition, any other structural anomalies were documented.

### 2.8. Data Quality Control

Before analysing the blood samples for *T. gondii* seropositivity, the test kits were calibrated by using the provided standards and controls. In addition, for each test run, positive and negative controls were included. Results were only considered valid if the controls and standard passed the test.

### 2.9. Data Management and Analysis

Data collected were recorded into a computer using Microsoft Office Excel 2007. Data was analysed with the STATA version 12 software (College Station, Texas, USA). Categorical variables were summarized as percentages and analysed by chi-square or Fisher's exact tests where appropriate. Continuous variables were summarized as mean (standard deviation) or median (interquartile range) where appropriate. Univariate and multivariate analyses were performed using a generalized linear model to determine the risk factors of *T. gondii* seropositivity. Risk factors with a *p* value less than 0.20 on univariate analysis were fitted into the multivariate analysis; risk ratios and 95% confidence intervals were noted. Risk factors with *p* values less than 0.05 were considered statistically significant.

## 3. Results

### 3.1. Sociodemographic Characteristics

The mean age of the enrolled children was 4.8 ± 3.5 months. Forty-eight (38.4%) were aged 2–4 months, and most of them 76/125 (60.8%) were male. Most of the infants 76/125 (60.8%) were from rural areas of the city, while in birth order 53/125 (42.4%) they lay between being second and fourth born (Table 1).

### 3.2. Clinical Characteristics

Out of 125 enrolled children, the mean occipital frontal circumference was 48.4 ± 6.8 cm and 36/125 (28.8%) infants had a history of neural tube defects. A history of fever and convulsions before the onset of hydrocephalus was observed in 28/125 (22.4%) and 22/125 (17.6%) infants, respectively (Table 2).

### 3.3. Seropositivity of Specific *T. gondii* Antibodies among Infants with Hydrocephalus

Out of 125 infants with hydrocephalus tested for *T. gondii-*specific antibodies, 29/125 (23.2%, 95% CI: 16.5–31.5) were found to be IgG seropositive while none of them was IgM seropositive.

### 3.4. Risk Factors Associated with *T. gondii-*Specific IgG Seropositivity among 125 Infants with Hydrocephalus

On bivariate analysis, a high birth order (RR = 1.3, 95% CI: 1.0–1.5, *p* = 0.004) was significantly associated with specific *T. gondii* IgG seropositivity (Table 3). In addition, consuming fish (RR = 1.6, 95% CI: 1.1–2.2, *p* = 0.008), and using different meat preparation methods (roasting, frying, deep frying, etc.) other than boiling meat (RR = 1.7, 95% CI: 1.1–2.6, *p* = 0.011) were significantly associated with specific *T. gondii* IgG seropositivity (Table 4). By multiple generalized linear model analysis, male gender (aRR = 1.1, 95% CI: 0.9–1.5, *p* = 0.049), consuming fish more than four times in a week (RR = 1.6, 95% CI: 1.2–2.3, *p* = 0.003), increasing birth order (aRR = 1.2, 95% CI: 1.0–1.5, *p* = 0.023), and using different methods of cooking other than boiling (aRR = 1.7, 95% CI: 1.1–2.5, *p* = 0.015) were found to predict specific *T. gondii* IgG seropositivity (Table 5).

### 3.5. Cranial Ultrasound Patterns

**Figure 2:**
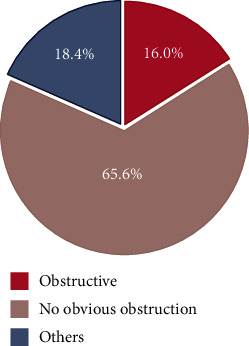
Overview of cranial ultrasound patterns in 125 infants with hydrocephalus.

**Figure 3:**
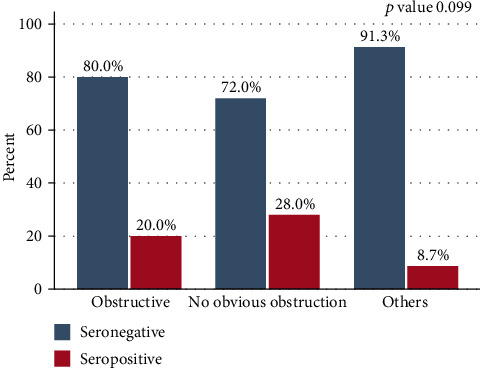
Cranial ultrasound patterns in seropositive and seronegative infants with hydrocephalus.

**Figure 4:**
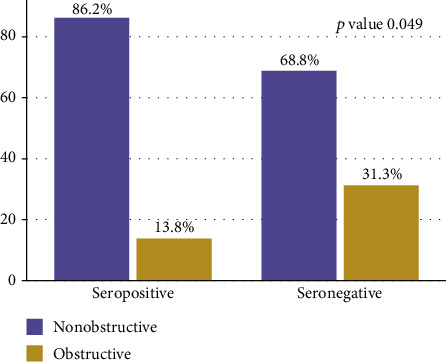
IgG seropositivity and cranial ultrasound patterns in infants with hydrocephalus.

Among 125 infants with hydrocephalus, the majority, 82/125 (65.6%), had no obvious obstruction while other patterns reported were obstructive (Dandy Walker cyst, Arnold Chiari malformation) (Figure 2). Further analysis showed that IgG seropositivity was likely to occur in infants with no obvious obstruction as compared to others; however, it was not statistically significant (Figure 3). Overall obstructive hydrocephalus was borderline significantly more common among *T. gondii* IgG-seronegative infants than in IgG-seropositive infants (31.3% [30/96] vs. 13.8% [4/29]; *p* = 0.049) (Figure 4).

Further analysis of 29 infants with hydrocephalus revealed that their median age was 2 (IQR 1–7) months, most of them were from rural areas 17/29 (58.6%), and 20/29 (69.0%) were male. The mean maternal age was 28.4 ± 7.3 years, and severe communicating hydrocephalus was present in 15/29 (51.7%) (Table 6).

## 4. Discussion

To the best of our knowledge, this is the first study to assess the association between the seropositivity of *T. gondii* antibodies and hydrocephalus among infants in Africa. In the present study, the seropositivity of specific IgG antibodies was 23.2% among infants with hydrocephalus which is significantly higher than in infants without hydrocephalus, when compared with the United States, Southeast Brazil, and France that reported a seropositivity of 0.01%, 0.1%, and 0.3%, respectively [18–20]. The possible explanation could be the endemicity of *T. gondii* in the study area whereby IgG seropositivity of 30.9% has been previously reported among pregnant women in the same setting [4, 13]. On the other hand, warm climatic conditions in the study area could explain the fact; a warm climate has been found to favour sporulation of oocysts elsewhere [21, 22].

In the current study, none of the infants was found to be IgM seropositive which is consistent with other studies where IgM detection was less than 0.01% [23]. This could be explained by the possibility that the infants in this study might have seroconverted while in the intrauterine environment, especially before the third trimester. Other possibilities for reactive IgG while IgM is nonreactive could be if the infection was acquired early in pregnancy, if maternal IgG is able to cross the placenta and suppress the IgM response in the fetus, or if the child was born prematurely with an immature immune system [24]. Moreover, in the current study, *T. gondii* IgG seropositivity was observed more frequently in neonates than in other age groups, even though the difference was not statistically significant. It has been established that the seropositivity of *T. gondii* tends to increase with an increase in age [25]. There are possibilities that most of the infants in the current study acquired these antibodies from their mother.

In the present study, the risk of being IgG seropositive was higher among male infants than among their female counterparts. However, it is generally documented that no gender predilection occurs with *T. gondii* seropositivity [26]. Further studies to explore the possibility of increased male gender susceptibility to *T. gondii* infection are warranted.

In this study, the risk of being IgG seropositive was higher among infants who were fifth born and above compared to their counterparts. This could be explained by the fact that an increase in the birth order is related with an increase in maternal age [27]. In the previous study by Mwambe et al., the risk of being *T. gondii* seropositive increased by 7% with a year increase in age [4]. Therefore, these infants were more likely to be born from mothers who were seropositive. This is further supported by the current study whereby mothers with fifth-born children and above were significantly older than those with a first-born child.

In the current study, consuming fish more than four times a week presented a significantly increased risk of being *T. gondii* IgG seropositive. This could be explained by the fact that fish is a staple food in Mwanza areas and is usually medium cooked (undercooked). There are limited studies that have investigated *T. gondii* in fish; therefore, more research is needed in this area [28, 29]. However, due to the small sample size, small number of participants reported to mainly consume fish, pork, and sheep and use other methods to prepare meat; therefore, these results should be interpreted considering this limitation. Outbreaks of toxoplasmosis associated with raw meat consumption have been reported elsewhere [30]. Studies have documented that different animals' meat such as sheep, cattle, beef, duck, pork, and goat could be common sources of *T. gondii* infection [30–32]. Other works have also reported that *T. gondii* is a common pathogen in fish and its presence in these animals may indicate the contamination of the aquatic environment by oocysts of the parasite [33, 34]. Further studies to establish relationship between toxoplasmosis and fish consumption around Lake Victoria zone are warranted.

In this study, infants whose mothers reported to prefer other methods of meat preparation than boiling, like frying and grilling, had significantly higher odds of being *T. gondii* IgG seropositive than those who preferred the boiling method. Consuming undercooked meat has been reported as a potential source of *T. gondii* infection as confirmed in the current study [35]. A previous study showed that a temperature of more than 70°C is required for the cyst to be destroyed which is not likely to be attained by methods like partial grilling in local settings [36].

Our results show that infants whose mothers reported symptoms suggestive of *T. gondii* infection during pregnancy were less likely to be *T. gondii* seropositive. This could be explained by the fact that the presence of these symptoms during pregnancy signifies an acute infection in the mother but not necessarily in the infant. During an acute infection, the predominant immunoglobulins are IgM, which do not cross the placenta. Of note, different studies have shown that more than half of the mothers could not recall their symptoms [37] and it is likely that these symptoms were nonspecific. Furthermore, this study did not capture information regarding treatment history of mothers to check the possibility of using some antimicrobials that can prevent transmission to fetus. However, in the study setting, there is no routine programme among pregnant women of screening for *T. gondii* and providing preventive treatment. Further studies to establish the relationship between the occurrence of *T. gondii* symptoms and congenital toxoplasmosis are warranted in this area.

In the current study, nonobstructive hydrocephalus was the most common pattern observed among infants who were IgG seropositive. This is consistent with studies in murine models which suggested that nonobstructive patterns can occur due to intraventricular or leptomeningeal inflammation, hindering cerebrospinal fluid reabsorption [38]. These findings are inconsistent though with a previous study which reported obstructive hydrocephalus to be a common feature among infants with congenital toxoplasmosis [39]. The differences could be attributed to the presence of severe hydrocephalus, the most common finding reported in the current study, which makes the assessment of congenital toxoplasma lesions by brain ultrasounds difficult as compared to brain computed tomography (brain CT scan) [17]. Further studies involving brain CT scan are warranted in this setting.

## 5. Study Limitation

A major limitation of this study was the inability to follow-up the trend in IgG antibody titers for at least two months which could confirm congenital toxoplasmosis. In addition, recall bias in obtaining retrospective information from the participants cannot be ruled out.

## 6. Conclusion and Recommendations

A significant proportion of infants with hydrocephalus have specific *T. gondii* IgG antibodies which is predicted by male gender, increase in birth order, and using other methods of cooking meat than boiling. Nonobstructive hydrocephalus patterns were commonly reported among infants who were *T. gondii* IgG seropositive. Emphasis should be put into the education of pregnant women regarding *T. gondii* risk factors, such as food preparation. In addition, there is a need to introduce serologic screening of *T. gondii* during pregnancy to identify infected women so that appropriate counselling and management can be provided. Moreover, routine fetal ultrasounds during pregnancy and head circumference measurements during Reproductive and Child Health (RCH) clinic visits should be performed for those infants who are at high risk of acquiring the infection, in order to detect hydrocephalus as early as possible.

## Figures and Tables

**Table 1:** Sociodemographic characteristics and other relevant characteristics among infants and their mothers.

Children characteristics	Number (*n*)	Percentage (%)
*Age group*		
Neonates	30	24.0
Early infancy	48	38.4
Late infancy	47	37.6
*Gender*		
Female	49	39.2
Male	76	60.8
*Birth order*		
1^st^ born	37	29.6
2^nd^-4^th^ born	53	42.4
>5^th^ born	35	28.0
*Residence*		
Urban	49	39.2
Rural	76	60.8
∗*Caregiver's level of education*		
Incomplete	33	26.4
Primary school	70	56.0
Secondary school	22	17.6
∗*Caregiver's employment*		
Peasants	84	67.2
Not employed	18	14.4
Employed	23	18.4
∗*Marital status*		
Not married	16	12.8
Married	109	87.2
∗*Maternal age*		
<35 years	115	92.0
>35 years	10	8.0

∗Mothers/guardians' characteristics.

**Table 2:** Clinical characteristics of 125 infants with hydrocephalus.

Characteristics	Number (*n*)	Percentage (%)
*Neural tube defect*		
No	89	71.2
Yes	36	28.8
*History of fever before HDC*		
No	97	77.6
Yes	28	22.4
*History of convulsions before HDC*		
No	103	82.4
Yes	22	17.6
*Developmental milestone*		
Delayed	60	48.0
Regressed	4	3.2
Up to date	61	48.8

**Table 3:** Sociodemographic characteristic risk factors associated with *T. gondii* seropositivity among 125 enrolled infants and their mothers.

Factors	Toxoplasma seroprevalence		Bivariate RR [95% CI]	*p* value
	IgG Pos *N* (%)	IgG Neg *N* (%)		
*Age*				
Neonates	8 (26.7)	22 (73.3)		
Early infancy	12 (25.0)	36 (75)	1.0 [0.8–1.2]	0.867
Late infancy	9 (19.2)	38 (80.9)	0.9 [0.8–1.1]	0.450
*Gender*				
Female	9 (18.4)	40 (81.6)		
Male	20 (26.3)	56 (73.7)	1.1 [0.9–1.3]	0.306
*Order of birth*				
2^nd^-4^th^ born	6 (11.3)	47 (88.7)		
1^st^ born	10 (27.0)	27 (73.0)	1.2 [1.0–1.3]	0.076
5^th^ born and above	13 (37.1)	22 (62.9)	1.3 [1.0–1.5]	0.004
*Residence*				
Rural	17 (22.4)	59 (77.6)		
Urban	12 (24.5)	37 (75.5)	1.0 [0.9–1.2]	0.785
∗*Education*				
Incomplete	10 (30.3)	23 (69.7)		
Primary	13 (18.3)	58 (81.7)	0.9 [0.7–1.1]	0.179
Secondary and above	6 (28.6)	15 (71.4)	0.9 [0.8–1.2]	0.884
∗*Occupation*				
Unemployed	4 (22.2)	14 (77.8)		
Peasants	18 (21.4)	66 (78.6)	1.0 [0.8–1.2]	0.943
Employed	7 (30.4)	16 (69.6)	1.1 [0.8–1.4]	0.540
∗*Marital status*				
Not married	4 (25.0)	12 (75.0)		
Married	25 (22.9)	84 (77.06)	1.0 [0.8–1.2]	0.856
∗*Maternal age*				
<35 yrs	25 (21.7)	90 (78.3)		
>35 yrs	4 (40.0)	6 (60.0)	1.2 [0.9–1.6]	0.190
*Maternal (S)*				
No	24 (26.1)	68 (73.9)		
Yes	5 (15.2)	28 (84.9)	0.9 [0.8–1.1]	0.202
*Animal keeping*				
No	9 (19.2)	38 (80.1)		
Yes	20 (25.6)	58 (74.4)	1.1 [0.9–1.2]	0.407
∗*Hand washing*				
No	3 (18.8)	13 (81.3)		
Yes	26 (23.9)	83 (76.2)	1.1 [0.8–1.3]	0.654
*Type of latrines*				
Latrines	19 (23.8)	61 (76.3)		
Bush/dustbin	10 (22.2)	35 (77.8)	1.0 [0.8–1.2]	0.847

∗Mothers/guardians' characteristics.

**Table 4:** Other risk factors associated with *T. gondii* transmission.

Factors	Toxoplasma seroprevalence		Bivariate RR [95% CI]	*p* value
	IgG Pos *N* (%)	IgG Neg *N* (%)		
*Contact with cats*				
No	8 (18.6)	35 (81.4)		
Yes	21 (25.6)	61 (74.4)	1.0 [0.9–1.3]	0.380
*Type of meat consumed*				
*Fish*				
No	25 (21.0)	94 (79.0)		
Yes	4 (66.7)	2 (33.3)	1.6 [1.1–2.2]	0.008
*Beef*				
No	8 (24.2)	25 (75.8)		
Yes	21 (22.8)	71 (77.2)	1.0 [0.8–1.2]	0.870
*Goat*				
No	22 (21.6)	80 (78.4)		
Yes	7 (30.4)	16 (69.6)	1.1 [0.9–1.3]	0.365
*Pork*				
No	27 (27.3)	94 (77.7)		
Yes	2 (50.0)	2 (50.0)	1.3 [0.9–2.0]	0.197
*Sheep*				
No	27 (22.3)	94 (77.7)		
Yes	2 (50.0)	2 (50.0)	1.3 [0.9–2.0]	0.197
*Meat preparation*				
Boiling	26 (21.5)	95 (78.5)		
Other methods	3 (75.0)	1 (25.0)	1.7 [1.1–2.6]	0.011
*Grill meat*				
Never	20 (26.0)	57 (74.03)		
Everyday/weekly	6 (20.7)	23 (79.3)	0.9 [0.8–1.1]	0.569
Monthly	3 (15.8)	16 (84.2)	0.9 [0.7–1.1]	0.350
*Undercooked vegetable*				
No	14 (20.9)	53 (79.1)		
Yes	15 (25.9)	43 (74.1)	1.1 [0.9–1.2]	0.514
*Boiling water*				
No	15 (19.7)	61 (80.3)		
Yes	14 (28.6)	35 (71.4)	1.1 [0.9–1.3]	0.255
*Gardening*				
No	16 (22.2)	56 (77.8)		
Yes	13 (24.5)	40 (75.5)	1.0 [0.9–1.2]	0.765
*Inedible foods*				
No	27 (22.9)	91 (77.1)		
Yes	2 (28.6)	5 (71.4)	1.0 [0.8–1.5]	0.731
*Crawling*				
No	27 (22.7)	92 (77.3)		
Yes	2 (33.3)	4 (66.7)	1.1 [0.8–1.6]	0.549
*Sibling with hydrocephalus*				
No	28 (22.6)	96 (77.4)		
Yes	1 (100)	0 (0)	2.1 [0.9–5.0]	0.066

**Table 5:** Risk factors associated with *T. gondii* seropositivity among infants with hydrocephalus and their mothers.

Factors	Toxoplasma seroprevalence		Bivariate RR [95% CI]	*p* value	aRR [95% CI]	*p* value
	IgG Neg *N* (%)	IgG Pos *N* (%)				
*Age*						
Neonates	22 (73.3)	8 (26.7)				
Early infancy	36 (75.0)	12 (25.0)	1.0 [0.8–1.2]	0.867	1.0 [0.8–1.2]	0.912
Late infancy	38 (80.9)	9 (19.2)	0.9 [0.8–1.1]	0.450	0.9 [0.7–1.0]	0.133
*Gender*						
Female	40 (81.6)	9 (18.4)				
Male	56 (73.7)	20 (26.3)	1.1 [0.9–1.3]	0.306	1.1 [0.9–1.5]	**0.049**
∗*Education*						
Incomplete	23 (69.7)	10 (30.3)				
Primary	58 (81.7)	13 (18.3)	0.9 [0.7–1.1]	0.179	0.9 [0.7–1.1]	0.211
Secondary and above	15 (71.4)	6 (28.6)	0.9 [0.8–1.2]	0.884	0.9 [0.8–1.2]	0.648
∗*Maternal age*						
<35 yrs	90 (78.3)	25 (21.7)				
>35 yrs	6 (60.0)	4 (40.0)	1.2 [0.9–1.6]	0.190	1.1 [0.9–1.5]	0.391
*Order of birth*						
2^nd^-4^th^ born	47 (88.7)	6 (11.3)				
1^st^ born	27 (73.0)	10 (27.0)	1.2 [1.0–1.3]	0.076	1.2 [1.0–1.4]	**0.038**
5^th^ born and above	22 (62.9)	13 (37.1)	1.3 [1.0–1.5]	0.004	1.2 [1.0–1.5]	**0.023**
*Types of meat consumed*						
*Fish*						
No	94 (79.0)	25 (21.0)				
Yes	2 (33.3)	4 (66.7)	1.6 [1.1–2.2]	0.008	1.6 [1.2–2.3]	**0.003**
*Pork*						
No	94 (77.7)	27 (27.3)				
Yes	2 (50.0)	2 (50.0)	1.3 [0.9–2.0]	0.197	1.1 [0.7–1.6]	0.801
*Sheep*						
No	94 (77.7)	27 (22.3)				
Yes	2 (50.0)	2 (50.0)	1.3 [0.9–2.0]	0.197	1.2 [0.8–2.0]	0.355
*Meat preparation*						
Boiling	95 (78.5)	26 (21.5)				
Other methods	1 (25.0)	3 (75.0)	1.7 [1.1–2.6]	0.011	1.7 [1.1–2.5]	**0.015**
∗*Maternal (S)*						
No	68 (73.9)	24 (26.1)				
Yes	28 (84.9)	5 (15.2)	0.9 [0.8–1.1]	0.202	0.8 [0.7–1.0]	**0.037**
*Sibling with hydrocephalus*						
No	96 (77.4)	28 (22.6)				
Yes	0 (0)	1 (100)	2.1 [0.9–5.0]	0.066	1.5 [0.6–3.7]	0.390

aRR = adjusted risk ratio. ∗Mothers/guardians' characteristics.

**Table 6:** Cranial ultrasound patterns among *T. gondii* IgG-seropositive infants.

Age (months)	Gender	Residence	Maternal age	Birth order	IgG titers	Brain USS
2	Male	Rural	18	1	1.94492	Severe communicating HDC
1	Male	Rural	40	7	5.13559	Moderate communicating HDC
1	Male	Rural	30	5	3.66525	Mild communicating HDC
9	Female	Rural	40	10	2.52119	Dandy Walker cysts
3	Male	Rural	17	1	1.53814	Severe communicating HDC
10	Male	Urban	31	2	7.40254	Lateral ventricle dilatation
4	Male	Rural	33	7	1.26871	Paraventricular hydrocephalus post infection
4	Female	Urban	26	1	9.11905	Severe communicating HDC
2	Female	Urban	30	5	2.44898	Mild communicating HDC
2	Male	Urban	25	4	1.4932	Severe communicating HDC
2	Male	Urban	35	1	1.62585	Lateral ventricle dilatation >> right side with epidural hematoma
1	Male	Urban	20	1	1.30952	Mild communicating HDC
2	Female	Rural	24	3	2.2381	Severe communicating HDC
2	Female	Urban	18	1	6.26531	Severe communicating HDC
1	Female	Urban	35	7	3.29932	Severe communicating HDC
9	Male	Rural	28	5	7.61225	Moderate communicating HDC
1	Male	Rural	20	1	3.07483	Severe communicating HDC
2	Male	Urban	18	1	4.49838	Severe communicating HDC
3	Male	Rural	16	1	2.26537	Dandy Walker cyst
1	Female	Rural	25	3	11.0356	Severe communicating HDC
10	Male	Urban	27	1	7.83495	Severe communicating HDC
1	Female	Rural	33	7	10.8641	Lateral ventricle dilatation
1	Female	Rural	35	4	5.22654	Severe communicating HDC
5	Male	Rural	38	6	2.884	Moderate communicating HDC
7	Male	Urban	30	5	1.656	Mild communicating HDC
12	Male	Rural	39	9	1.252	Severe communicating HDC
7	Male	Urban	31	2	3.408	Severe communicating HDC
11	Male	Rural	32	7	3.12	Lateral ventricle dilatation
11	Male	Rural	29	5	3.432	Severe communicating HDC

## Data Availability

All data collected have been used for the current article. All data are available on request to the Director of Research & Innovations.
